# iTRAQ-based comparative proteomic analysis of differences in the protein profiles of stems and leaves from two alfalfa genotypes

**DOI:** 10.1186/s12870-020-02671-2

**Published:** 2020-09-29

**Authors:** Hao Sun, Jie Yu, Fan Zhang, Junmei Kang, Mingna Li, Zhen Wang, Wenwen Liu, Jiaju Zhang, Qingchuan Yang, Ruicai Long

**Affiliations:** 1 Institute of Animal Sciences, Chinese Academy of Agricultural Sciences, Beijing, 100193 China; 2 Key Laboratory of Animal Nutrition and Feed Science in South China, Ministry of Agriculture and Rural Affairs/ Guangdong Key Laboratory of Animal Breeding and Nutrition, Institute of Animal Science, Guangdong Academy of Agricultural Sciences, Guangzhou, 510640 Guangdong China

**Keywords:** Alfalfa, Proteomics, Stem, Leaf, Lignin synthesis, Photosynthesis

## Abstract

**Background:**

To explore the molecular regulatory mechanisms of early stem and leaf development, proteomic analysis was performed on leaves and stems of F genotype alfalfa, with thin stems and small leaves, and M genotype alfalfa, with thick stems and large leaves.

**Results:**

Based on fold-change thresholds of > 1.20 or < 0.83 (*p* < 0.05), a large number of proteins were identified as being differentially enriched between the M and F genotypes: 249 downregulated and 139 upregulated in stems and 164 downregulated and 134 upregulated in leaves. The differentially enriched proteins in stems were mainly involved in amino acid biosynthesis, phenylpropanoid biosynthesis, carbon fixation, and phenylalanine metabolism. The differentially enriched proteins in leaves were mainly involved in porphyrin and chlorophyll metabolism, phenylpropanoid biosynthesis, starch and sucrose metabolism, and carbon fixation in photosynthetic organisms. Six differentially enriched proteins were mapped onto the porphyrin and chlorophyll metabolism pathway in leaves of the M genotype, including five upregulated proteins involved in chlorophyll biosynthesis and one downregulated protein involved in chlorophyll degradation. Eleven differentially enriched proteins were mapped onto the phenylpropanoid pathway in stems of the M genotype, including two upregulated proteins and nine downregulated proteins.

**Conclusion:**

Enhanced chlorophyll synthesis and decreased lignin synthesis provided a reasonable explanation for the larger leaves and lower levels of stem lignification in M genotype alfalfa. This proteomic study aimed to classify the functions of differentially enriched proteins and to provide information on the molecular regulatory networks involved in stem and leaf development.

## Background

In contrast to animals, land plants constantly produce new tissues and organs to complete their life cycle, and the formation of stems and leaves is a crucial aspect of plant growth and development. The plant shoot, which includes leaves, stems, and flowers, differentiates from the shoot apical meristem (SAM) [1]. Leaves, which originate from the SAM [2], are the central organs of photosynthesis and photoperception. In addition, they are the first barriers for plant defense, protecting plants against photochemical damage from UV rays, maintaining plant mechanical properties, and preventing damage from biotic and abiotic factors [3]. As for the stem, plant stem cells are dynamic structures composed of polysaccharides, phenolic compounds, and proteins; stems connect plant organs and transport nutrition through the xylem and phloem [4]. The plant stem is not only essential for maintaining plant height and rigidity but also responds to environmental stress through the release of signaling molecules [5].

Alfalfa (*Medicago sativa* L.) is a widely cultivated perennial legume forage with high nutritional value [6]. Compared with the gradual decline in nutritional value of the alfalfa stem during plant maturation, the nutritional value of alfalfa leaves remains high throughout the growing season [7]. During its growth and development, alfalfa’s nutritional value changes as the ratio of stem to leaf increases [8]. The ratio of stem to leaf is a relevant index that reflects the quality of alfalfa. Tender plants have a high leaf yield, high crude protein content, and high nutritional value. As the plants grow and age, the leaf ratio decreases, dry matter content increases, cellulose content increases, cell walls gradually thicken, and organic sugars increase. The decrease in relative food value of the stem is due to changes in the deposition of cell wall components [9]. To elucidate the differences in cell wall concentration and composition of stems of two alfalfa genotypes, several candidate genes were identified in transcript profiling [10]. With declining quality, the leaf is an essential source of nutrition for alfalfa and directly affects the quality of alfalfa hay [6, 11, 12].

In recent years, an extensive collection of genes involved in alfalfa stem development and leaf morphogenesis have been identified. However, the molecular regulation of stem development and leaf morphogenesis is an intricate biological process that has yet to be fully revealed. Classical genetic approaches are insufficient to elucidate the molecular regulation mechanisms of leaf morphogenesis and stem development, and proteomics has been used as a complementary approach for protein functional identification [13]. The impressive set of proteomics tools and approaches for studying differentially enriched proteins can provide insight into the mechanisms responsible for differences in leaf and stem architecture. Proteome analysis of different stem regions has shown that a subset of proteins involved in cellulose biosynthesis is significantly enriched in intermediate and mature basal regions, resulting in higher cellulose and lignin contents [14].

Here we report on a proteomic analysis of leaves and stems from F genotype alfalfa, with thin stems and small leaves (denoted FS and FL), and M genotype alfalfa, with thick stems and large leaves (denoted MS and ML). In contrast to a study of molecular mechanisms of leaf and stem development in the same genetic background published previously [10, 14, 15], here leaf and stem samples were collected from two alfalfa genotypes with different genetic backgrounds selected from a panel of low and high forage yields. This study was designed to identify differentially enriched proteins related to leaf morphogenesis and stem development, thereby further exploring the regulatory mechanisms of early stem and leaf development.

## Results

### Phenotypic differences between alfalfa genotypes

**Fig. 1:**
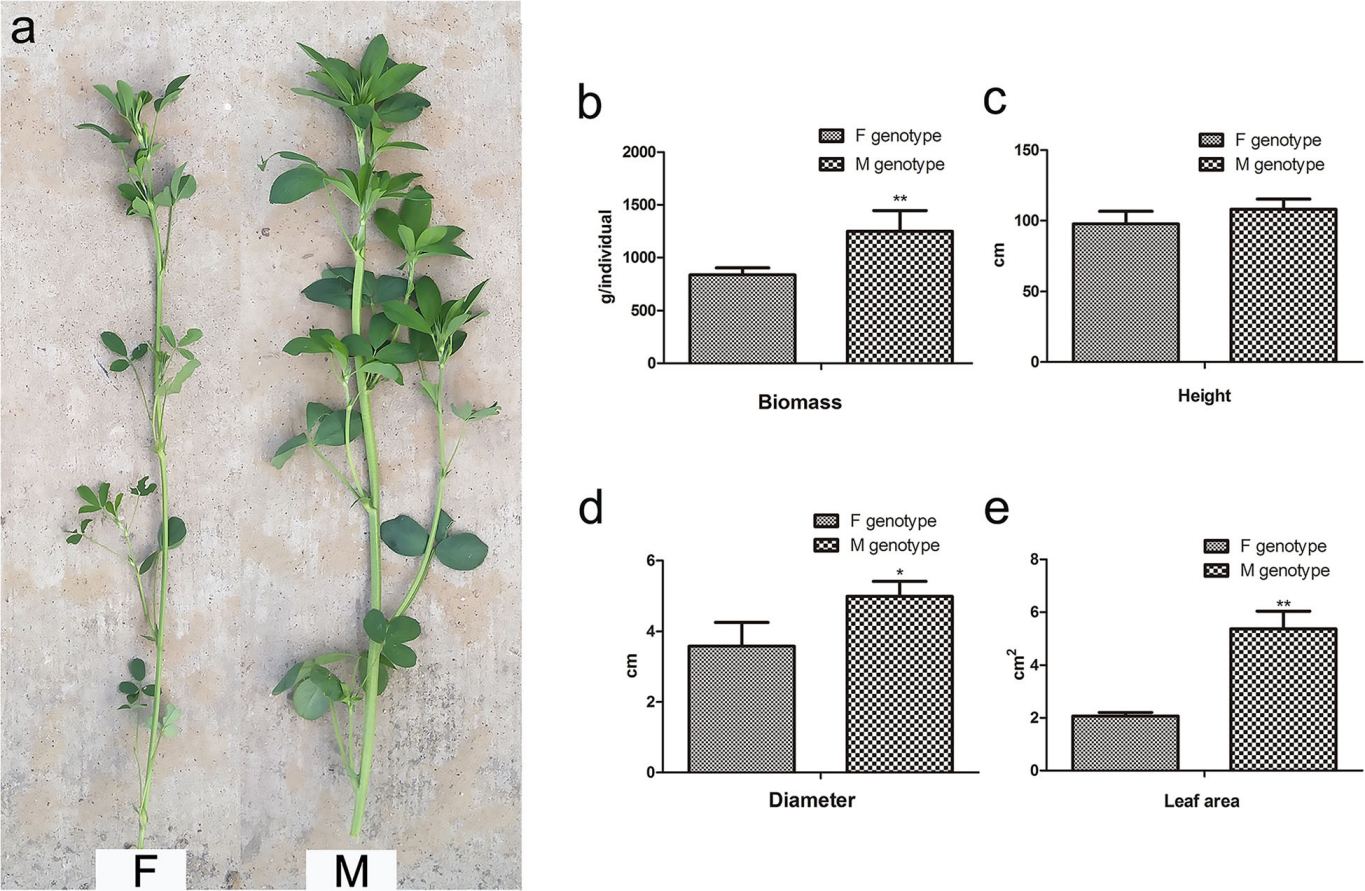
Phenotypic identification of two alfalfa genotypes in 2017. **a**. The phenotypes of two alfalfa genotypes; **b**–**e**. The agronomic traits of two alfalfa genotypes: biomass, height, diameter, and leaf area. Three biological replicates were analyzed, error bars denote SDs, and data were analyzed by one-way ANOVA (*p < 0.05, ** *p* < 0.01, Duncan’s multiple range test)

There were significant phenotypic differences between the F and M genotypes (Fig. 1a), especially in biomass. Individual plant biomass in M genotype alfalfa was significantly higher than that in F genotype alfalfa, consistent with differences in stem diameter and leaf area (Fig. 1b). The biomass of the M genotype was 48.7% higher than that of the F genotype, and the leaf area of the M genotype was 2.6 times higher than that of the F genotype. However, although the height of the M genotype alfalfa was slightly greater than that of the F genotype alfalfa, there was no significant difference in height between the two genotypes. Near-infrared reflectance spectroscopy (NIRS) was used to measure the nutritional values of the two genotypes. The M genotype had greater nutritional value than the F genotype, including higher crude protein concentrations and lower cellulose and lignin contents (Supplementary Fig. S1).

### Quantitative identification of proteins in alfalfa stems and leaves using iTRAQ

**Fig. 2:**
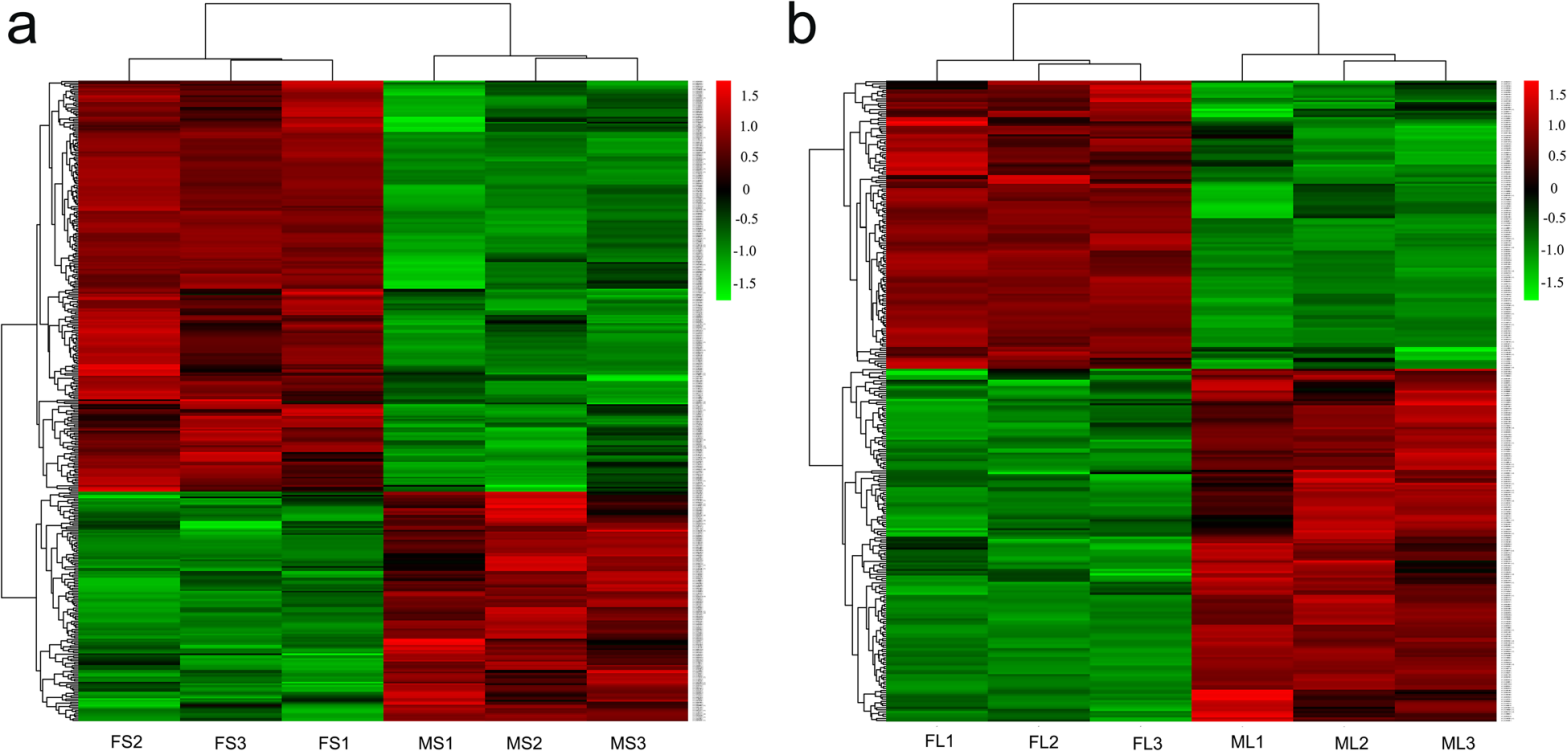
Hierarchical clustering analysis of differentially enriched proteins identified in stems (**a**) and leaves (**b**). Dataset clustering was performed in R (version 3.2.2) after normalization of the expression abundance values. Each colored cell represents the average spot quantity, and the color scale indicates the fold-change of the DEPs. FS: stem of F genotype, MS: stem of M genotype; FL: leaf of F genotype, ML: leaf of M genotype

**Fig. 3:**
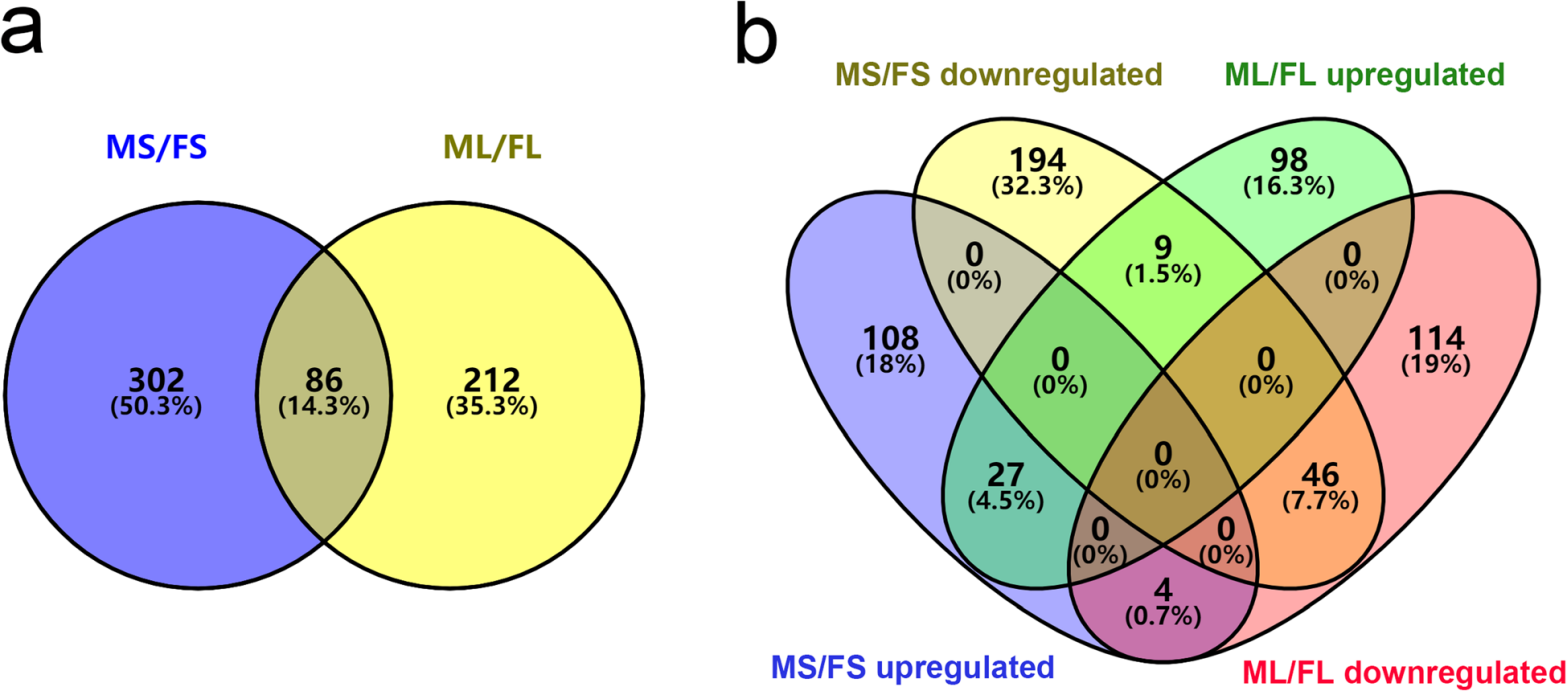
Venn diagram analysis of common DEPs identified in leaves and stems of two alfalfa genotypes. **a**, the overlapping portion denotes 86 common DEPs identified in stems and leaves of two alfalfa genotypes. **b**, Upregulated and downregulated DEPs identified and quantified in ML/FL and MS/FS. “Upregulated” indicates higher protein abundance in former one of the two contrast genotype alfalfa. “Downregulated” indicates lower protein abundance in former one of the two contrast genotype alfalfa

We obtained 14,315 unique peptides, including 3784 identified proteins (Supplementary Table 1 and 2). A fold-change threshold of > 1.20 or < 0.83 (*p* < 0.05) was used to identify differentially enriched proteins (DEPs) between FS and MS and between FL and ML. Using these criteria, 388 DEPs were identified in stems, including 249 downregulated and 139 upregulated DEPs (Fig. 2a, Supplementary Table 3). Likewise, 298 DEPs were identified in leaves: 164 downregulated and 134 upregulated (Fig. 2b, Supplementary Table 4). Compared with F genotype alfalfa, eighty-six DEPs (14.3%) were identified in stems and leaves, including twenty-seven upregulated and forty-six downregulated (Fig. 3a–b). Whereas, the remaining thirteen DEPs showed the opposite trend in stems and leaves. Furthermore, four DEPs were upregulated in stems, while downregulated in leaves. On the contrary, nine DEPs were upregulated in leaves and downregulated in stems (Fig. 3b).

### Functional categorization of differentially enriched proteins

**Fig. 4:**
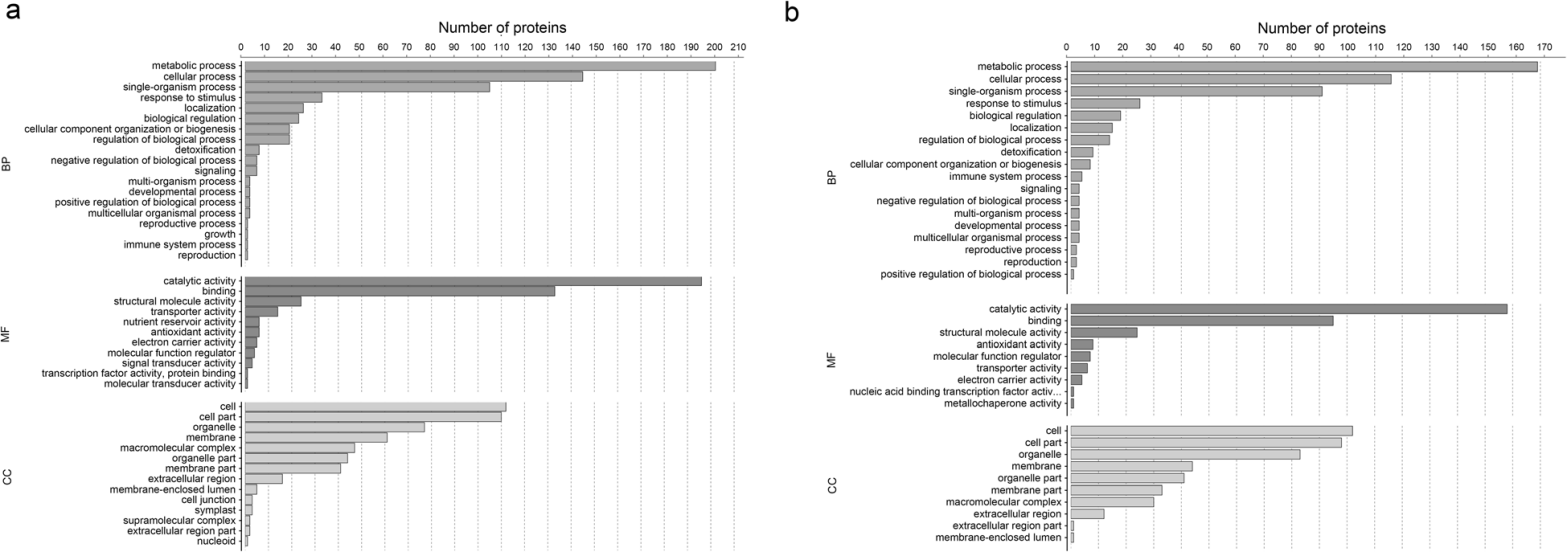
GO classification of the identified DEPs in stems (**a**) and leaves (**b**). Results are summarized under three main GO categories: biological process (BP), cellular component (CC), and molecular function (MF)

GO enrichment was performed to identify the biological functions of the DEPs, categorizing them according to their biological process, cellular component, and molecular function (Supplementary Tables 5 and 6). The primary biological process categories in stems and leaves were metabolic process, cellular process, response to stimulus, and biosynthetic regulation. The most abundant cellular component categories were cell, cell part, and organelle, and the most abundant molecular function categories in stems (Fig. 4a) and leaves (Fig. 4b) were catalytic activity, binding, structural molecule activity, and transport activity.

**Fig. 5:**
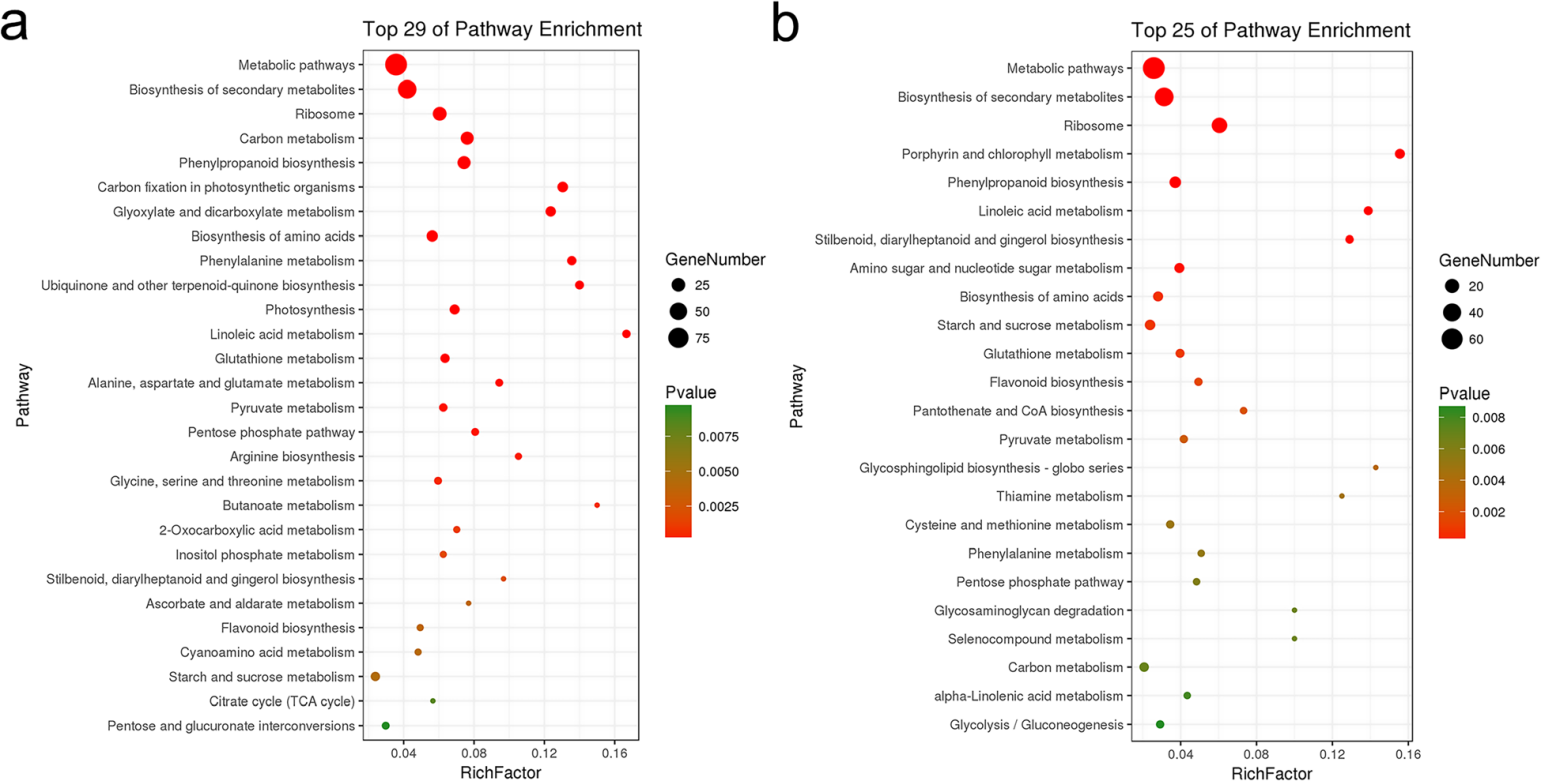
Functional significant enrichment of DEPs identified in stems (**a**) and leaves (**b**). The point size indicates the number of annotated DEPs, and the color depth indicates the *p*-value (*p* ≤ 0.01) associated with enrichment. Details of significantly enriched DEPs are provided in Supplementary Tables 9 and 10

KEGG analysis was performed to gain insight into the biochemical pathways of the identified DEPs. The 388 DEPs in stems were mapped to 82 pathways, and the 298 DEPs in leaves were mapped to 76 pathways (Supplementary Tables 7 and 8). In addition to metabolic pathway, biosynthesis of secondary metabolites, and ribosome, stems also showed significant enrichment in 25 additional pathways, including carbon metabolism, phenylpropanoid biosynthesis, carbon fixation in photosynthetic organisms, glyoxylate and dicarboxylate metabolism, biosynthesis of amino acids, phenylalanine metabolism, ubiquinone and other terpenoid-quinone biosynthesis, photosynthesis, linoleic acid metabolism, glutathione metabolism, and alanine, aspartate and glutamate metabolism (Fig. 5a). Likewise, leaves were significantly enriched in 21 pathways, including porphyrin and chlorophyll II metabolism, phenylpropanoid biosynthesis, and linoleic acid metabolism were significantly enriched in leaves (Fig. 5b). According to the KEGG pathway bubble map, identified proteins (*p* < 0.01) primarily mapped to 28 sub-types in stems and 24 sub-types in leaves (Supplementary Tables 9 and 10).

### Differences in enriched metabolic pathways of stems and leaves

KEGG enrichment analysis of these proteins revealed significant overlap in enriched pathways: 61 pathways were enriched in both stems and leaves. Ten of these pathways were significantly co-enriched (*p < 0.01*), and these were mainly associated with carbon metabolism; phenylpropanoid biosynthesis; biosynthesis of amino acids; pentose phosphate pathway; starch and sucrose metabolism; pyruvate metabolism; stilbenoid, diarylheptanoid and gingerol biosynthesis; and flavonoid biosynthesis. The following pathways were specifically enriched in stems: carbon fixation in photosynthetic organisms; glyoxylate and dicarboxylate metabolism; ubiquinone and other terpenoid-quinone biosynthesis; photosynthesis; alanine, aspartate and glutamate metabolism; arginine biosynthesis; glycine, serine and threonine metabolism; butanoate metabolism; 2-oxocarboxylic acid metabolism; inositol phosphate metabolism; ascorbate and aldarate metabolism; cyanoamino acid metabolism; citrate cycle (TCA cycle); and pentose and glucuronate interconversions. Likewise, the following pathways were specifically enriched in leaves: porphyrin and chlorophyll metabolism; amino sugar and nucleotide sugar metabolism; pantothenate and COA biosynthesis; glycosphingolipid biosynthesis; thiamine metabolism; cysteine and methionine metabolism; selenocompound metabolism; glycosaminoglycan degradation; alpha-linolenic acid metabolism; and glycolysis / gluconeogenesis.

**Fig. 6:**
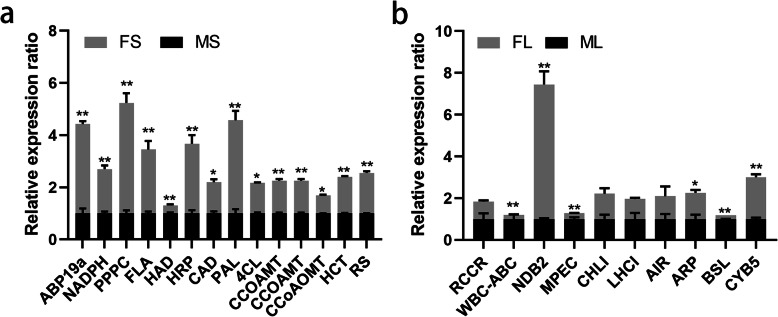
qRT–PCR analysis of mRNA transcripts associated with randomly selected DEPs from stems (**a**) and leaves (**b**). FS: stem (F), MS: stem (M); FL: leaf (F), ML: leaf (M). The expression of each gene was calculated relative to the β-actin reference gene using the 2^−△△CT^ method. Three biological replicates were analyzed, error bars denote SDs, and data were analyzed by one-way ANOVA (* *p* < 0.05, ** *p* < 0.01, Duncan’s multiple range test)

To further confirm the proteomic results, qRT-PCR was used to examine the mRNA expression of randomly selected proteins using specific primers (Supplementary Table 11). Compared with FS, thirteen DEPs (including auxin-binding protein ABP19a (ABP19a), NAD(P)H:quinone oxidoreductase, phosphoenolpyruvate carboxylase (PPPC), fasciclin domain protein, horseradish peroxidase-like protein (HRP), phenylalanine ammonia-lyase-like protein (PAL), cinnamyl alcohol dehydrogenase (CAD), shikimate/quinate hydroxycinnamoyl-transferase (HCT), riboflavin synthase alpha chain, 4-coumarate: CoA ligase-like protein (4CL-like), and caffeic acid O-methyltransferase (CCOAMT) were upregulated and caffeoyl-CoA 3-O-methyltransferase (CCoAOMT) and HAD-family hydrolase IIA) were identified and downregulated in MS (Fig. 6a). Furthermore, although the mRNA levels of CCOAMT and HAD-family hydrolase IIA were inconsistent with their protein levels, the mRNA expression profiles of eleven genes were consistent with their protein levels in stems (Fig. 6a, Supplementary Tables 3 and 4). In addition, compared with FL, ten DEPs (including light-harvesting complex I chlorophyll A/B-binding protein (LHCI), auxin-repressed/dormancy-associated protein (ARP), white-brown-complex ABC transporter family protein (WBC-ABC), red chlorophyll catabolite reductase (RCCR), auxin-induced in root cultures protein (AIR), cytochrome b5-like heme/steroid-binding domain protein (CYB5), and NAD(P) H dehydrogenase B2 (NDB2) were downregulated in FL. Magnesium-protoporphyrin IX monomethyl ester cyclase (MEPC), bark storage-like protein (BSL), and magnesium chelatase subunit ChlI (CHLI)) were significantly enriched and upregulated in ML. What’s more, the mRNA abundances of nine genes were consistent with their protein levels, while the mRNA abundances of two genes (LHCI and CHLI) were opposite to their protein levels in leaves (Fig. 6b, Supplementary Table 5).

## Discussion

### Involvement of lignin biosynthesis in the regulation of stem development and secondary growth

As internodes elongate, stem lignification gradually deepens. Increasing stem maturity accelerates stem lignification and subsequently increases the stem/leaf ratio, resulting in decreased digestibility. Previous studies have shown that the maturity of the stem affects the digestibility of forage, and improving digestibility is one of the primary goals of alfalfa breeding [16]. Therefore, in order to monitor changes in the digestibility of alfalfa, recent research has focused on the expression of key genes involved in the phenylpropanoid biosynthesis pathway [17, 18]. Cell wall synthesis, including the synthesis of cellulose, hemicellulose, and lignin, is a complex biological process. Cellulose biosynthesis is performed by a membrane-bound rosette structure of which sucrose synthase is an integral component [19]. The presence of lignin, a complex phenolic polymer formed from three alcohol monomers (coumarin, coniferol, and myrosinol), is the main reason for reduced alfalfa digestibility [20]. Lignin biosynthesis requires the participation of a variety of enzymes, and lignin is one of the essential products of the phenylpropanoid metabolic pathway. The specific biosynthesis of monolignols begins with the production of phenylalanine in the shikimate pathway [21, 22] and continues with the formation of 3-p-hydroxyphenyl, guaiacyl, and syringyl units and finally the synthesis of lignin [23].

**Fig. 7:**
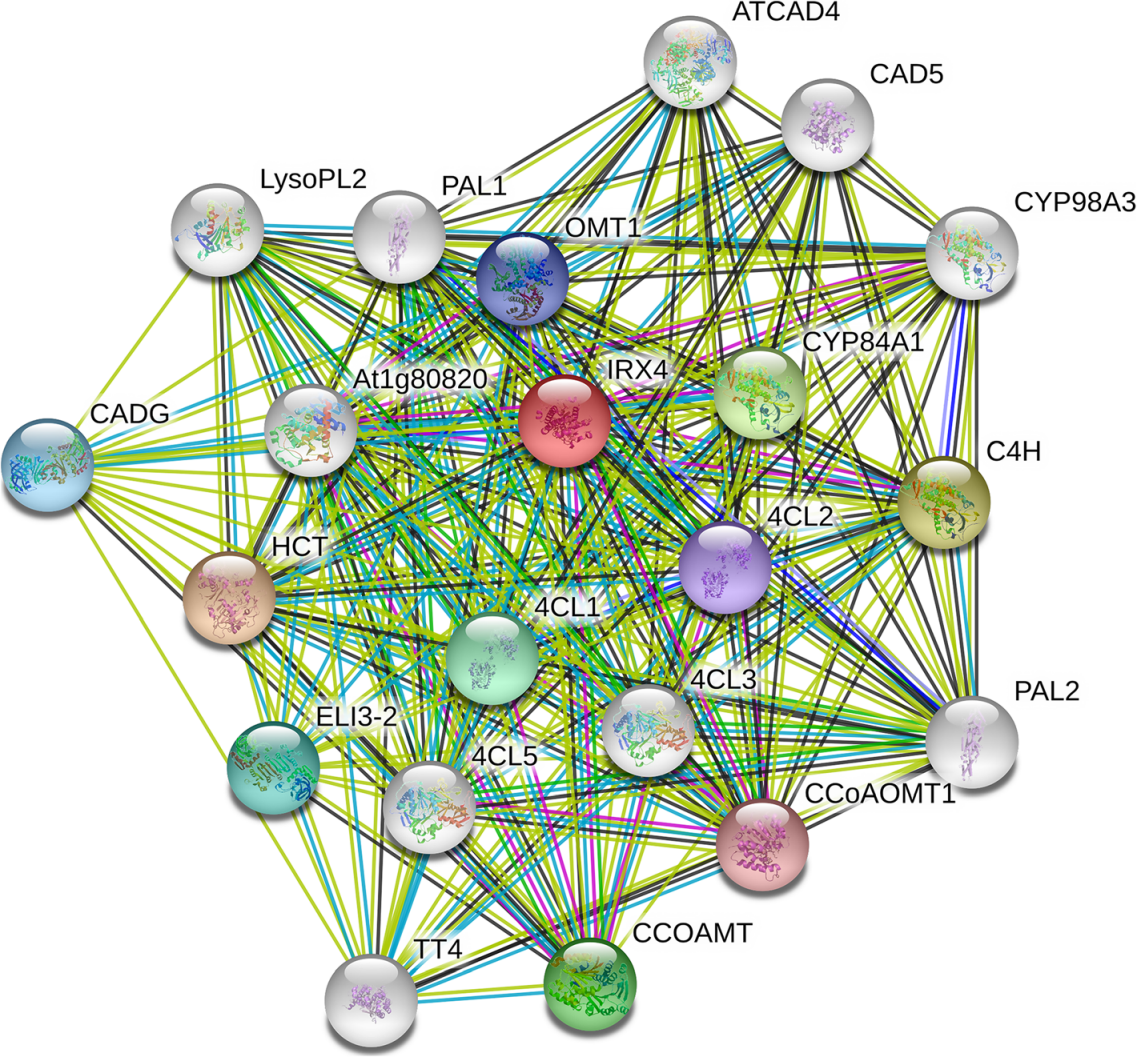
Protein–protein interaction network of DEPs related to phenylpropanoid biosynthesis. The network was generated using the STRING database (https://string-db.org/) with *Arabidopsis thaliana* IRX4 (cinnamoyl-CoA reductase 1) selected as input. The network was expanded by an additional ten proteins using the ‘More’ button in the STRING interface, and the confidence cutoff for interaction links was set to ‘highest’ (0.900). The protein interaction network was mapped with *A. thaliana* as a reference

**Fig. 8:**
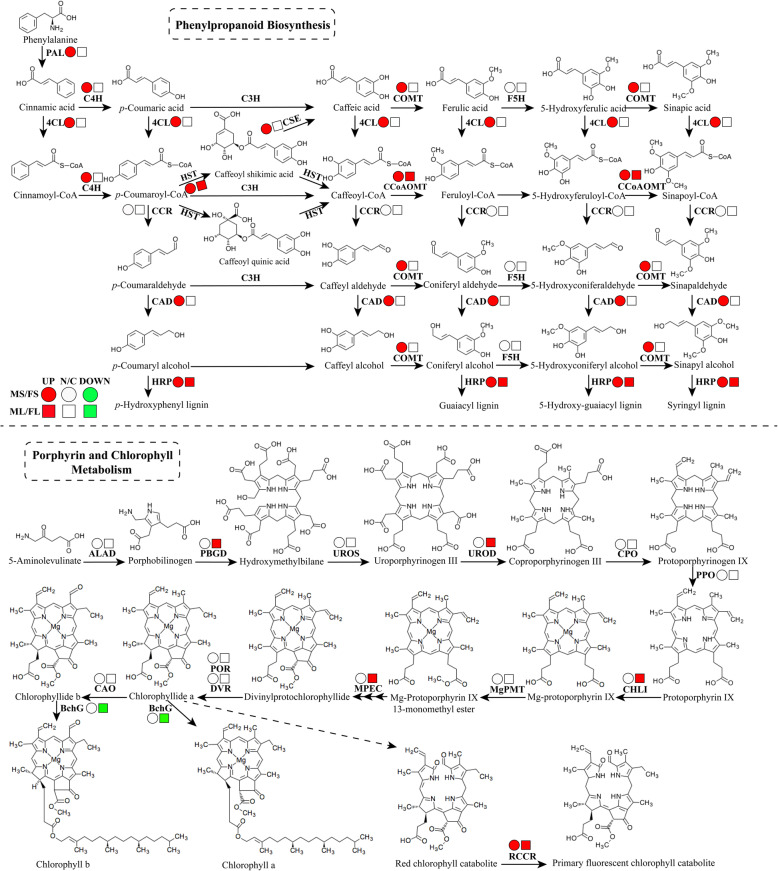
Abundance changes in proteins related to phenylpropanoid biosynthesis and porphyrin and chlorophyll metabolism. a. Overview of phenylpropanoid biosynthesis pathways in *Medicago sativa* L. Enzyme abbreviations are as follows. Phenylalanine ammonia-lyase-like protein (PAL), cytochrome P450 family cinnamate 4-hydroxylase (C4H), caffeic acid O-methyltransferase (COMT), 4-coumarate: CoA ligase-like protein (4CL), monoglyceride lipase-like protein (MGL), HXXXD-type acyl-transferase family protein (HCT), caffeoyl-CoA 3-O-methyltransferase (CCoAOMT), cinnamyl alcohol dehydrogenase-like protein (CAD), horseradish peroxidase-like protein (HRP), glycoside hydrolase family 1 protein (GH1), and cytochrome P450 family monooxygenase (F5H); b. Overview of porphyrin and chlorophyll metabolism pathways in *M. sativa* L. Enzyme abbreviations are as follows. Porphobilinogen deaminase (PBGD), uroporphyrinogen decarboxylase (UPOD), bacteriochlorophyll synthase (BchG), red chlorophyll catabolite reductase (RCCR), magnesium-chelatase subunit ChlI, and magnesium-protoporphyrin IX monomethyl ester cyclase (MPEC). The circles represent proteins identified in stems, whereas the squares represent proteins identified in leaves. Red indicates upregulation, white indicates no significant changes, and green indicates downregulation in M genotype alfalfa

An interaction network of 21 proteins was involved in phenylpropanoid biosynthesis (Fig. 7), and most of the key enzymes of phenylpropanoid biosynthesis were identified in the current study. In total, 11 DEPs were mapped onto the phenylpropanoid pathway (Fig. 8a), including phenylalanine ammonia-lyase-like protein (PAL), cytochrome P450 family cinnamate 4-hydroxylase (C4H), caffeic acid O-methyltransferase (CCoAMT), 4-coumarate: CoA ligase-like protein (4CL), monoglyceride lipase-like protein (MGL), HXXXD-type acyl-transferase family protein (HCT), caffeoyl-CoA 3-O-methyltransferase (CCoAOMT), cinnamyl alcohol dehydrogenase-like protein (CAD), horseradish peroxidase-like protein (HRP), glycoside hydrolase family 1 protein (GH1), and cytochrome P450 family monooxygenase (F5H). Compared with MS, nine of these proteins were significantly upregulated in FS, whereas HXXXD-type acyl-transferase family protein (HCT) and cytochrome P450 family monooxygenase (F5H) were downregulated. These expression changes provide a reasonable explanation for the higher levels of stem lignification in the F genotype (Supplementary Fig. S1).

Phenylalanine ammonia-lyase-like protein (PAL), an enzyme associated with lignification in primary and secondary tissues, catalyzes the deamination of phenylalanine to initiate phenylpropanoid metabolism [24]. The expression of PAL varies coordinately with condensed tannins (CTs) accumulation during the primary to secondary growth transition in stems, and PAL is mainly expressed in non-lignifying cells of stems, leaves, and roots [25]. In the current study, PAL was downregulated in MS compared with FS, consistent with the maturation of the stem (Fig. 6a), suggesting that PAL played an essential role in phenylpropanoid metabolism as well as in stem development. A previous study has reported that both coumarate 3-hydroxylase (C3H) and C4H are involved in the early steps of monolignol biosynthesis and exert a negative effect on stem digestibility [18]. In the current study, the content of C4H was significantly lower in MS than in FS (Fig. 6a), consistent with lower monolignol biosynthesis and higher stem digestibility in MS.

CAD catalyzes the final step in monolignol synthesis and is therefore a crucial enzyme for the synthesis of S-, G-, and H-lignin [26]. 4CL plays a part in the biosynthesis of lignin monomers, particularly guaiacyl (G) lignin [27], and there is an overlap in the expression patterns of 4CL family genes [28]. In the current study, CAD, 4CL, and C4H were enriched abundantly in the phenylpropanoid biosynthesis pathway. Furthermore, all of these proteins exhibited consistently lower protein and transcript levels in MS than in FS (Fig. 6a), consistent with greater lignin accumulation in FS. Previous studies have reported that the downregulation of CCoAOMT and CCoAMT leads to lower lignin levels and a reduction in G units [29]. In the current study, CCoAOMT and CCOAMT were significantly downregulated in MS relative to FS (Fig. 6a), consistent with lignin biosynthesis in MS.

Previous studies have reported that shikimate/quinate HCT plays an essential role in lignin biosynthesis, and there is a strong correlation between HCT accumulation and lignin properties [30]. In the current study, protein and transcript levels of shikimate/quinate HCT were lower in MS than in FS, consistent with lower lignin accumulation in MS. Moreover, four lipid-transfer proteins, eight serine proteases, and six fasciclin domain proteins (including 3 fasciclin-like arabinogalactan protein, FLA) were also enriched in stems, and these proteins have been reported to participate in cell wall maturation and secondary growth [31, 32].

Taking all of the genes mentioned above into consideration, DEPs were involved in lignin biosynthesis and the regulation of stem development and secondary growth. Although the M genotype had a thicker stem (Fig. 1a), the expression of DEPs associated with lignin synthesis was lower in M than in F, suggesting that M accumulated less lignin. This indicates that the M genotype may have higher biological yields and a higher nutritional value than the F genotype (Fig. 1b, Supplementary Fig. S1).

### Differences in metabolism-related DEPs between leaves of two alfalfa genotypes

Leaves originate from the SAM and ultimately become flat organs specialized to facilitate light capture and photosynthesis. Leaf morphogenesis, which gives rise to a wide variety of sizes and shapes, is an intricate process that is regulated by many genes from multiple pathways [33–36]. Early leaf development is arbitrarily divided into three stages: the initiation of the leaf primordium, the establishment of leaf adaxial-abaxial polarity, and the expansion of the leaf blade [37, 38]. Previously, KANADI and YABBY transcription factors have been reported to be responsible for the development of abaxial tissues [39–42]. Initial asymmetric leaf development is regulated by polar YABBY expression [43]. Furthermore, gain-of-function alleles of KANADI and YABBY3 (YAB3) result in radial abaxialized organs [40, 41] and abaxial tissue differentiation, respectively [39, 42].

Leaf development is regulated by internal genetic mechanisms and external environmental cues. Phytohormones, especially auxin, regulate the entire process of leaf development [44]. Plant auxin homeostasis is mainly maintained by three processes: de novo IAA biosynthesis, IAA degradation, and IAA conjugation/deconjugation. In addition, IAA carboxyl methyltransferase has been reported to have an essential role in the regulation of auxin homeostasis through the conversion of IAA to methyl-IAA ester (MeIAA) [45]. In the current study, indole-3-acetaldehyde oxidase, which is involved in the biosynthesis of IAA, was lower in ML than in FL, suggesting that less auxin was produced in ML [46]. In addition, the trend in indole-3-acetaldehyde oxidase levels was consistent with that of ARP and AIR (Fig. 6b), which are involved in auxin homeostasis and play vital roles in leaf development.

Photosynthesis is a process used by plants and other organisms to convert light energy into chemical energy that can later be released to fuel the organisms’ activities. Photosystems are functional and structural units of protein complexes involved in photosynthesis that together carry out the primary photochemistry of photosynthesis: the absorption of light and the transfer of energy and electrons. LHCI influences the capture of light energy by photosystem II, which is the key step in the light reactions [47]. The chloroplast NAD (P) H dehydrogenase (NDH) complex, as an electron donor, mediates photosystem I (PSI) cyclic and chlororespiratory electron transport [48, 49]. Efficient operation of NDH requires supercomplex formation via minor LHCI in *Arabidopsis*, and both play essential roles in photosynthesis [50]. In the current study, LHCI and NDH were downregulated in ML relative to FL (Fig. 6b). The reduced protein abundance may have resulted in a decreased photosynthetic rate per unit leaf area in ML. However, compared with F genotype alfalfa, larger leaf area in M genotype alfalfa (Fig. 1b) might have compensated for the decrease of photosynthetic rate in ML, which provided a reasonable explanation for higher leaf biomass in ML.

Green plants obtain most of their energy from sunlight via photosynthesis by chloroplasts (including chlorophylls a and b), which gives them their green color. Interestingly, DEPs associated with carbon fixation metabolism and photosynthesis were significantly enriched in leaves, which supply energy for plant development. Chlorophyll, the primary pigment in plant leaves, mainly participates in photosynthesis, and chlorophyll biosynthesis plays an essential role in leaf development. The insertion of magnesium into the chlorophyll molecule is primarily controlled by the activity of magnesium chelatase subunit CHLI, which performs a critical step in the chlorophyll biosynthetic pathway [51, 52]. In the current study, six DEPs were mapped onto the porphyrin and chlorophyll metabolism pathway, including porphobilinogen deaminase (PBGD), uroporphyrinogen decarboxylase (UPOD), bacteriochlorophyll synthase (BchG), red chlorophyll catabolite reductase (RCCR), CHLI, and magnesium-protoporphyrin IX monomethyl ester cyclase (MPEC), which were significantly enriched in leaves (Fig. 8b). Moreover, both CHLI and MPEC [53] were significantly upregulated in ML relative to FL, suggesting that chlorophyll biosynthesis and photosynthetic efficiency were higher in ML (Fig. 6b). In addition, a higher content of granule-bound starch synthase I in ML also suggested the accumulation of more photosynthetic products [54], consistent with higher biomass in ML than in FL.

Chlorophyll degradation occurs during leaf senescence, the final stage of leaf development that is regulated by transcription factors and receptor kinases through signal perception and transduction [55, 56]. Recent research has shown that chlorophyllase, magnesium-chelating substance, and RCCR participate in chlorophyll breakdown [57, 58]. In the current study, RCCR, a major inducer of cell death [59], had consistently lower protein and transcript levels in ML than in FL (Fig. 6b), probably resulting in RCCR accumulation in FL. Enhanced chlorophyll degradation likely further reduced photosynthetic rate, contributing to the differences in biomass observed between the alfalfa genotypes.

### Comparative proteomics analysis of DEPs in leaves and stems

Substance synthesis and energy metabolism provide an essential nutrient supply for plants and are indispensable for the completion of normal growth and development. Previous studies have reported that carbohydrate metabolism directly affects plant growth status [60]. Interestingly, carbon metabolism and energy metabolism showed similar trends in stems and leaves. Likewise, the largest group of DEPs between the alfalfa genotypes was also related to metabolism in leaves and stems. DEPs involved in glycolysis/gluconeogenesis, the pentose phosphate pathway, and pyruvate metabolism were identified in leaves (Fig. 5b). Similarly, a large number of DEPs involved in the pentose phosphate pathway, the citrate cycle (TCA cycle), and glyoxylate and dicarboxylate metabolism were identified in stems of the alfalfa genotypes (Fig. 5a). In addition, most of the DEPs identified and upregulated in MS and ML were involved in carbohydrate metabolism and amino acid biosynthesis, indicating that primary metabolism was enhanced to facilitate leaf and stem development and promote increased biomass in the M genotype.

Stem and leaf tissue provide necessary nutrients and material reserves for flower development at the budding stage. In the current study, linoleate 13S-lipoxygenase 2–1 and seed linoleate 9S-lipoxygenase involved in the inositol phospholipid signaling pathway were identified in stems and leaves. Previous studies have shown that various components of the inositol phospholipid signaling system participate in vacuolar changes during pollen development and in vesicle transport during pollen tube growth [61]. The decreased protein abundance of linoleate 13S-lipoxygenase 2–1 and seed linoleate 9S-lipoxygenase in the M genotype suggested that this genotype could maintain longer vegetative growth and avoid earlier reproductive growth, which was conducive to the accumulation of more biomass.

Anthocyanin biosynthesis plays an indispensable role in pollen development, especially for alfalfa that is cross-pollinated. To avoid self-pollination in alfalfa, floral pigmentation is conducive to attracting insects and transmitting pollen. Dihydroflavonol 4-reductase (DFR), an essential enzyme for anthocyanin biosynthesis that catalyzes the reaction of dihydroflanovol to leuco- cyanidin (−delphynidin and -pelargonidin) [62], was significantly downregulated in M genotype alfalfa, further demonstrating that M genotype alfalfa could maintain longer vegetative growth. Moreover, as the final storage form of photosynthetic products, dark storage protein was upregulated and enriched in the M genotype, providing a further explanation for the increased biomass of the M genotype.

The biological functions of 302 DEPs identified only in stems and 212 DEPs identified only in leaves were further analyzed. In terms of lignin biosynthesis and phenylalanine metabolism, although the DEPs were identified in leaves and stems, almost all the enzymes associated with phenylalanine pathways were significantly enriched in stems, whereas few accumulated in leaves, suggesting that lignin synthesis and phenylalanine metabolism mainly play a role in stem development. However, the differences in biomass between the two genotypes derived from differences in photosynthetic efficiency. Several DEPs involved in porphyrin and chlorophyll metabolism (including PBGD, UPOD, BchG, ChlI, and MPEC) and granule-bound starch synthase I were only identified in the leaf proteome and were upregulated in ML, providing a reasonable explanation for the higher biomass of the M genotype.

## Conclusions

Leaf and stem development, responsible for plant morphology and establishment, is a complicated process that runs throughout the whole life history of plants. Especially for alfalfa, there is always a paradox between yield and quality. To obtain high yields and high-quality alfalfa, it is imperative to study the molecular mechanisms that regulate alfalfa stem and leaf development. In the current study, six DEPs were mapped onto the porphyrin and chlorophyll metabolism pathway in ML, including five upregulated proteins involved in chlorophyll biosynthesis and one downregulated protein involved in chlorophyll degradation. At the same time, eleven DEPs were mapped onto the phenylpropanoid pathway in MS, including two upregulated proteins and nine downregulated proteins. Enhanced chlorophyll synthesis and decreased lignin synthesis provided a reasonable explanation for the larger leaves and lower levels of stem lignification in M genotype alfalfa. This analysis of stem and leaf protein expression profiles at the budding stage contributes to our understanding of the molecular mechanisms that underlie alfalfa stem and leaf development.

## Methods

### Plant material

Two alfalfa genotypes, F genotype alfalfa with thin stems and small leaves derived from the local variety “Cangzhou” (CF000735) (F) and M genotype alfalfa with thick stems and large leaves derived from the cultivated variety Zhongmu No.1 (CF032020) (M), were individually selected from a panel of low and high forage yields, respectively. The plant material was collected and cloned by our laboratory, and the approximate dormancy classification of the two alfalfa genotypes was level 3. During the early branching stage in spring 2014, one plant each was chosen from the F and M genotype alfalfa and cloned by cuttage propagation. The cloned plants were then planted in the field of the CAAS research station in Langfang, Hebei Province (39.59°N, 116.59°E). Three individual plants from the ten cloned plants obtained from each genotype were randomly selected for further analysis. Agronomic traits of three randomly selected individual plants were measured at the budding stage of the first stubble under natural conditions in 2017: aboveground biomass, plant height, leaf area, and stem diameter (Fig. 1a). Aboveground biomass was obtained by measuring the dry weight of three individual plants, and height was obtained by measuring the highest branch of three individual plants. The diameters of five randomly selected branches at the shoot base of each plant were measured after harvesting. Leaf areas were obtained by measuring the terminal leaflet of trifoliate leaves located at the third node of three individual plants. Due to differences in the initial flowering period of the two materials, branches were sampled at the same physiological stage (i.e., when the flower buds had not yet opened and were still in bud). Stem and leaf samples were collected simultaneously at the budding stage. Samples of stems (approximately the first three internodes from the stem apex) and leaves (including petioles and stipules and located within the first three internodes) were collected from three individual cloned plants from each genotype and used for proteome and gene expression analysis. The M genotype stem, M genotype leaves, F genotype stem, and F genotype leaves were labeled MS, ML, FS, and FL, respectively. All samples were immediately frozen in liquid nitrogen and stored at − 80 °C prior to protein and RNA extraction. Random samples were collected from the two alfalfa genotypes and dried to constant weight at 105 °C for 48 h. The dry samples were crushed into pieces, sieving for 1 mm, and stored under conditions of low temperature, dark and heat insulation. The quality of the dried samples was analyzed with a near infrared spectrometer system (FOSS NIR System 5000, FOSS, Denmark) over the 1100–2500 nm wavelength range at 2 nm wavelength intervals. Measurements were performed under stable working conditions at room temperature (25 °C), and WinI-SI III calibration software was used. Each sample was loaded and scanned three times, the average value was taken, and the spectral data was recorded in the form of log (1/R). Under the same working parameters and conditions, composition analysis (including crude protein, NDF, ADF, and lignin) of the two alfalfa genotypes was performed with same calibration software. All experiments were conducted with three biological replicates. The experimental design and technical process are presented as a technical roadmap in Supplementary Fig. S2.

### Protein extraction and purification

Proteins were extracted and purified according to our previous publication [63]. The samples were ground to a powder in liquid nitrogen and mixed thoroughly with lysis buffer (7 M urea, 2 M thiourea, 4% CHAPS, 50 ml protease inhibitor cocktail). The mixture was vigorously vortexed and ultrasonicated for 60 s (0.2 s on /2 s off) with an amplitude of 22%. The extract was incubated at room temperature for 30 min and then centrifuged at 15,000×g at 10 °C for 1 h. The supernatant was transferred to a new micro-centrifuge tube that contained 4× volume of precooled 10% (w/v) trichloroacetic acid (TCA)/acetone, and proteins were precipitated overnight at − 20 °C. The samples were centrifuged at 13,000×g for 10 min at 4 °C, and the precipitate was rinsed with precooled acetone and centrifuged. Protein concentration was measured using a Bradford assay kit (Bio-Rad Laboratory), and bovine serum albumin (BSA) was used to generate the standard curve.

### iTRAQ labelling and LC-MS/MS analysis

Proteins were digested using the filter-aided sample preparation (FASP) workflow as follows [64]. For each sample, DTT was added to 200 μg of protein sample to a final concentration of 25 mM at 60 °C for 1 h. The mixture was alkylated with 50 mM iodoacetamide at room temperature for 10 min, and then centrifuged at 12,000×g for 20 min. 100 μL of dissolution buffer was added, and the mixture was centrifuged at 12,000×g. The filtrate was finally discarded, and the protein samples were digested with trypsin overnight (Promega, Madison, WI, USA) at 37 °C. The peptides were labelled with isobaric tags from the iTRAQ Reagent-8plex Multiplex Kit (AB Sciex, USA) according to the manufacturer’s recommended procedure [65]. The labelled peptide mixtures were then pooled and dried by vacuum centrifugation. 100 μl aliquots of labeled samples were loaded at a flow rate of 0.7 ml/min. The components obtained from the reverse phase separation with high pH were redissolved in 20 μl 2% methanol and 0.1% formic acid, followed by centrifugation at 12,000×g. 10 μl of supernatant was loaded at a flow rate of 0.35 ml/min for 15 min. Separation velocity was set at 300 nl/min.

### Protein identification and quantification

Database searches were performed as described in our previous publication [63]. The extraction of tandem mass spectra was performed on the ProteoWizard (version 3.0.8789). The raw mass data was analyzed using the Mascot server (Matrix Science, London, UK; version 2.6.0) to retrieve the *Medicago truncatula* database (version 4.0, 57,693 entries) according to the description in our previous publication [63]. Label Based Quantitation (iTRAQ, TMT, SILAC, etc.) of identified peptides and proteins was performed with Scaffold Q^+^ (version Scaffold_4.6.2, Proteome Software Inc., Portland, OR) as described in a previous publication [65]. Moreover, peptide and protein identification were established according to our previous publication [63]. Protein probabilities were assigned by the protein prophet algorithm [66]. Proteins that contained similar peptides and could not be differentiated based on MS/MS analysis alone were grouped to satisfy the principles of parsimony. As described in the previous study, normalization was iterated over intensity [67]. Means were used for differential analysis. Spectral data were log-transformed, pruned of those that matched multiple proteins or were missing a reference value, and weighted by an adaptive intensity weighting algorithm. Additionally, a fold-change threshold of > 1.20 or < 0.83 (*p* < 0.05) was used to identify differentially enriched proteins based on Student’s *t*-test.

### qRT–PCR analysis

Total RNA was isolated with the SV total RNA extraction system (Promega, Madison, WI, USA) according to the manufacturer’s instructions. First-strand cDNA was synthesized using the Prime Script™ RT reagent kit (Takara, Beijing, China). qRT**–**PCR was performed on an ABI Prism 7300 detection system (Applied Biosystems) using the Takara TB Green™ Premix Ex Taq™ II (Tli RNaseH Plus) kit (Takara, Beijing, China). The mRNA transcription levels of eleven DEPs (including red chlorophyll catabolite reductase (XP_003617849.2), white-brown-complex ABC transporter family protein (XP_003630284.1), pectinesterase/pectinesterase inhibitor (XP_003622699.1), NAD(P) H dehydrogenase B2 (XP_013455573.1 (+ 1)), magnesium-protoporphyrin IX monomethyl ester cyclase (XP_003624856.1), magnesium-chelatase subunit ChlI (XP_003593716.1), light-harvesting complex I chlorophyll A/B-binding protein (XP_013450965.1), auxin-induced in root cultures protein (XP_003626890.1), auxin-repressed/dormancy-associated protein (XP_003593620.1), bark storage-like protein (XP_013462016.1), and cytochrome b5-like heme/steroid-binding domain protein (XP_003627519.1)) identified in leaves and thirteen DEPs (including 4-coumarate: CoA ligase-like protein (XP_003604127.1), auxin-binding protein ABP19a (XP_003627273.1), NAD(P)H: quinone oxidoreductase (XP_003615608.1), horseradish peroxidase-like protein (XP_003602461.1), phosphoenolpyruvate carboxylase (XP_003597124.1), phenylalanine ammonia-lyase-like protein (XP_003591877.1), fasciclin domain protein (XP_003597210.2), cinnamyl alcohol dehydrogenase (XP_003628601.1), HAD-family hydrolase IIA (XP_003608097.1), caffeoyl-CoA 3-O-methyltransferase (XP_003607927.1), riboflavin synthase alpha chain (XP_013443595.1), shikimate/quinate hydroxycinnamoyl-transferase (XP_013454529.1), and caffeic acid O-methyltransferase (XP_003602396.1)) identified in stems were measured, and the β-actin gene (mRNA, EU664318.1) was used as an internal reference. Relative gene expression was quantified using the 2^−△△CT^ method [68]. The analysis was performed with three biological replicates, and all primer sequences are provided in Supplementary Table 11, and the raw data was listed in Supplementary Table 12 and 13.

### Bioinformatic analysis of proteins

Functional category analysis was performed with BLAST2GO software (http://www.geneontology.org) [69]. The KEGG database (http://www.genome.jp/kegg/pathway.html) was used to obtain current information on biochemical pathways and molecular interactions [70]. After expression abundances were standardized, hierarchical clustering analysis was performed in R (version 3.2.2). A protein–protein interaction network of the DEPs involved in phenylpropanoid biosynthesis was generated using the STRING database (http://string-db.org) [71]. Gene expression data were analyzed for statistical significance using a one-way ANOVA (* indicates *p* < 0.05, ** indicates *p* < 0.01, Duncan’s multiple range test) in SPSS 21.0.

## Supplementary information

**Additional file 1: Figure S1.** The nutritional parameters of M and F genotype alfalfa.

**Additional file 2: Figure S2.** The technical roadmap of this work.

**Additional file 3: Table S1.** The total identified proteins by iTRAQ in MS and FS.

**Additional file 4: Table S2.** The total identified proteins by iTRAQ in ML and FL.

**Additional file 5: Table S3.** The differentially enriched proteins identified using iTRAQ in MS and FS.

**Additional file 6: Table S4.** The differentially enriched proteins identified using iTRAQ in ML and FL.

**Additional file 7: Table S5.** The GO annotation analysis of the differentially enriched proteins in MS and FS.

**Additional file 8: Table S6.** The GO annotation analysis of the differentially enriched proteins in ML and FL.

**Additional file 9: Table S7.** The KEGG enrichment analysis of the differentially enriched proteins in MS and FS.

**Additional file 10: Table S8.** The KEGG enrichment analysis of the differentially enriched proteins in ML and FL.

**Additional file 11: Table S9.** The significant KEGG enrichment analysis of the differentially enriched proteins in MS and FS.

**Additional file 12: Table S10.** The significant KEGG enrichment analysis of the differentially enriched proteins in ML and FL.

**Additional file 13: Table S11.** The primer sequences used in this study.

**Additional file 14: Table S12.** The raw data of qRT-PCR in stems.

**Additional file 15: Table S13.** The raw data of qRT-PCR in leaves.

## Data Availability

The data supporting the results of this article are included within the article and the provided supplementary files. The proteomics raw data was submitted to iProX database (project ID: IPX0002423000).

## Abbreviations

iTRAQ: Isobaric tags for relative and absolute quantification
NIRS: Near-infrared reflectance spectroscopy
NDB2: NAD(P) H dehydrogenase B2
PAL: Phenylalanine ammonia-lyase-like protein
C4H: Cytochrome P450 family cinnamate 4-hydroxylase
COMT: Caffeic acid O-methyltransferase
4CL: 4-Coumarate: CoA ligase-like protein
MGL: Monoglyceride lipase-like protein
HCT: HXXXD-type acyl-transferase family protein
CCoAOMT: Caffeoyl-CoA 3-O-methyltransferase
CAD: Cinnamyl alcohol dehydrogenase-like protein
HRP: Horseradish peroxidase-like protein
GH1: Glycoside hydrolase family 1 protein
F5H: Cytochrome P450 family monooxygenase
PBGD: Porphobilinogen deaminase
UPOD: Uroporphyrinogen decarboxylase
BchG: Bacteriochlorophyll synthase
RCCR: Red chlorophyll catabolite reductase
MPEC: magnesium-protoporphyrin IX monomethyl ester cyclase
BSL: Bark storage-like protein
ARP: Auxin-repressed/dormancy-associated protein
AIR: Auxin-induced in root cultures protein
