# Supplementary figures and images for: iTRAQ-based comparative proteomic analysis of differences in the protein profiles of stems and leaves from two alfalfa genotypes
Source: BMC Plant Biol. 2020 Sep 29;20:447. doi: 10.1186/s12870-020-02671-2 (PMC7525974; doi:10.1186/s12870-020-02671-2)

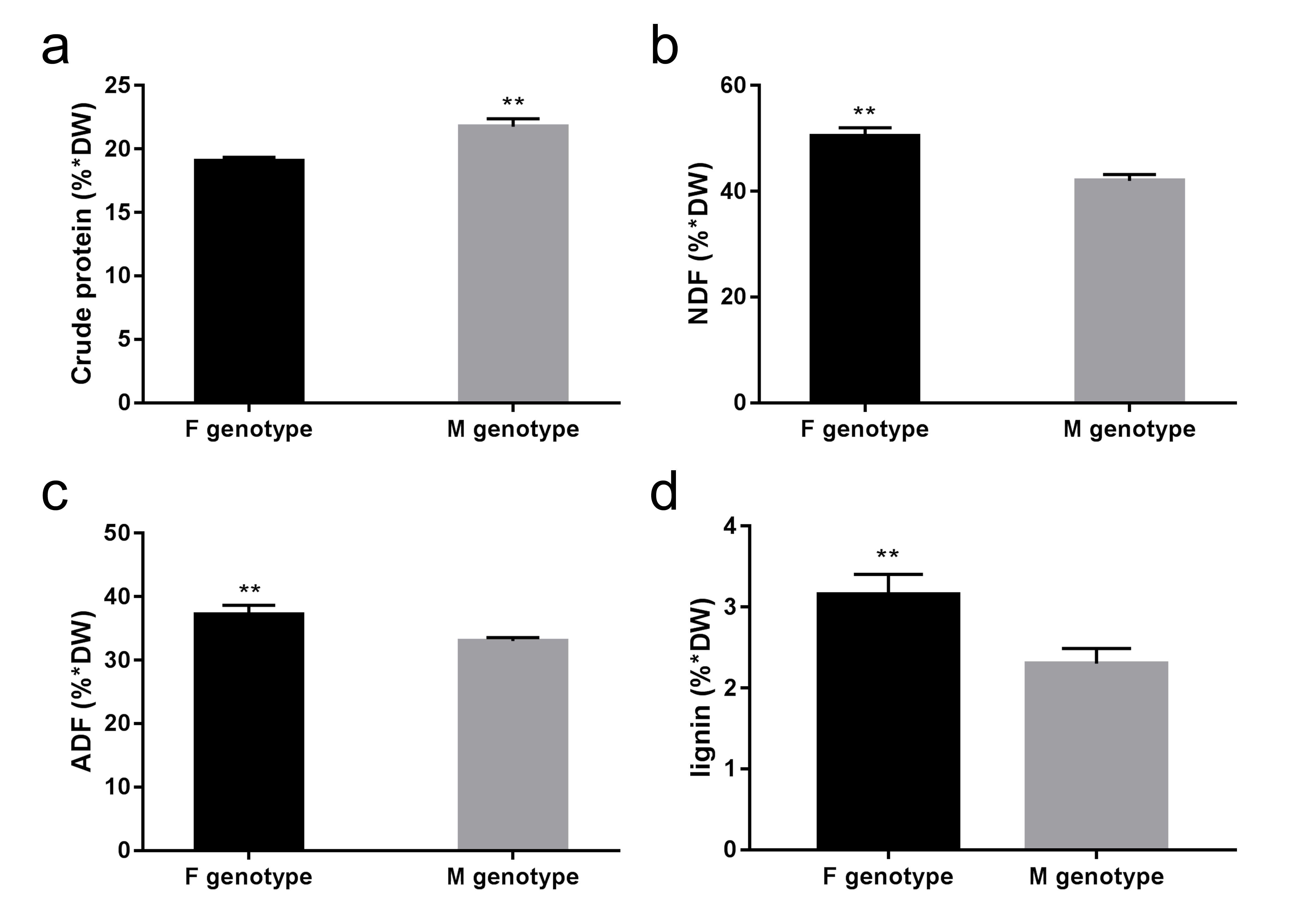

Supplement: Supplementary file 1 — Additional file 1: Figure S1. The nutritional parameters of M and F genotype alfalfa. [file 12870_2020_2671_MOESM1_ESM.tif]

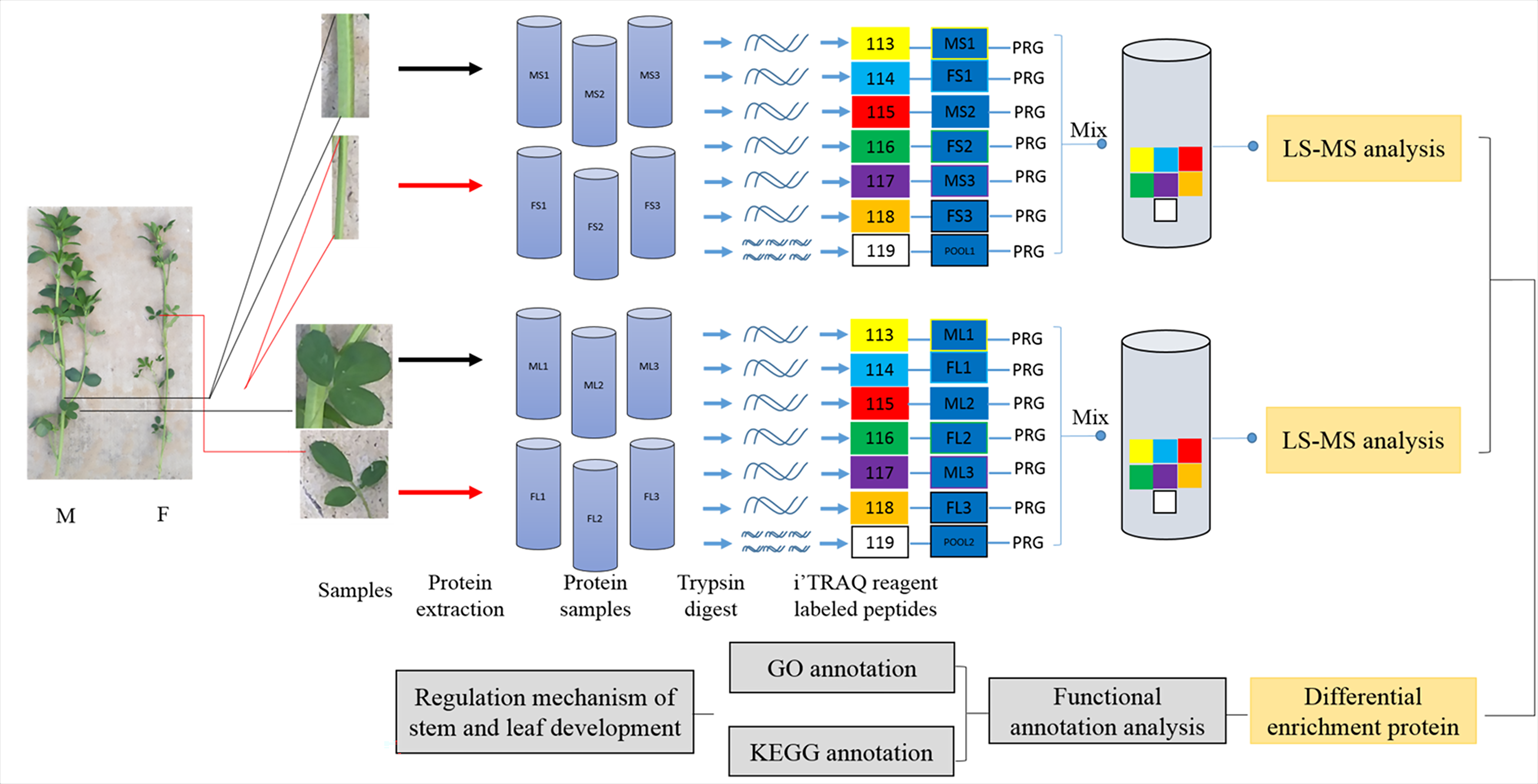

Supplement: Supplementary file 2 — Additional file 2: Figure S2. The technical roadmap of this work. [file 12870_2020_2671_MOESM2_ESM.tif]
